# Supplementary material for: Nitrate-functionalized patch confers cardioprotection and improves heart repair after myocardial infarction via local nitric oxide delivery
Source: Nat Commun. 2021 Jul 23;12:4501. doi: 10.1038/s41467-021-24804-3 (PMC8302626; doi:10.1038/s41467-021-24804-3)
Supplement: Supplementary file 1 — Supplementary information [file 41467_2021_24804_MOESM1_ESM.pdf]

Supplementary information for

Nitrate-functionalized patch confers cardioprotection and improves heart repair after myocardial infarction via local nitric oxide delivery

Dashuai Zhu[1,2,#], Jingli Hou[3,#], Meng Qian[1,#], Dawei Jin[4], Tian Hao[1], Yanjun Pan[4], He Wang[1], Shuting Wu[4], Shuo Liu[2], Fei Wang[1], Lanping Wu[5], Yumin Zhong[6], Zhilu Yang[7], Yongzhe Che[2], Jie Shen[8], Deling Kong[1], Meng Yin[4,*], Qiang Zhao[1,9,*]

[1] State key Laboratory of Medicinal Chemical Biology, Key Laboratory of Bioactive Materials (Ministry of Education), College of Life Sciences, Nankai University, Tianjin 300071, China
[2] School of Medicine, Nankai University, Tianjin 300071, China
[3]Tianjin Key Laboratory on Technologies Enabling Development of Clinical Therapeutics and Diagnostics, School of Pharmacy, Tianjin Medical University, Tianjin 300070, China
[4] Department of Cardiothoracic Surgery, Shanghai Children's Medical Center, School of Medicine, Shanghai Jiao Tong University, Shanghai 200127, China.
[5] Department of Cardiac Ultrasound, Shanghai Children's Medical Center, School of Medicine, Shanghai Jiao Tong University, Shanghai 200127, China.
[6] Diagnostic Imaging Center, Shanghai Children's Medical Center, School of Medicine, Shanghai Jiao Tong University, Shanghai 200127, China.
[7] Key Laboratory of Advanced Technology for Materials of Education Ministry, School of Materials Science and Engineering, Southwest Jiaotong University, Chengdu, 610031, China.
[8] College of Pharmacy, Nankai University, Tianjin 300071, China
[9] Zhengzhou Cardiovascular Hospital and 7th People's Hospital of Zhengzhou, Zhengzhou, Henan Province, China

# Zhu D, Hou J, and Qian M equally contributed to this study.
* Corresponding author. E-mail: qiangzhao@nankai.edu.cn or yinmengmdphd@163.com

Supplementary Table 1. Summary on structural parameters and mechanical properties of the electrospun cardiac patches. Data are expressed as mean±SD of at least five repeated measurements.

Measurement	PCL-patch	NO-patch
Fiber size(μm)	0.85±0.35	0.69±0.23
Pore size(μm)	4.81±1.86	3.42±0.93
Young’s modulus(MPa)	8.98±1.08	7.95±1.35
Stress at max(MPa)	5.80±0.54	4.75±0.38
Strain at break(%)	199.68±19.22	188.82±15.42

Supplementary Table 2. Primers used for Real-time quantitative PCR (qPCR).

Primer	Sequence
IL10-F	GCTGCCTTCAGTCAAGTGAA
IL10-R	GGCATCACTTCTACCAGGTA
Arg1-F	AAGAAA AGGCCGATTCACCT
Arg1-R	CACCTCCTCTGCTGTCTTCC
IL-1β-F	CACCTCTCAAGCAGAGCACAG
IL-1β-R	GGGTTCCATGGTGAAGTCAAC
TNF-α-F	TACTCCCAGGTTCTCTTCAAGG
TNF-α-R	GGAGGCTGACTTTCTCCTGGTA
Gapdh-F	ATGACTCTACCCACGGCAAG
Gapdh-R	CTGGAAGATGGTGATGGGTT

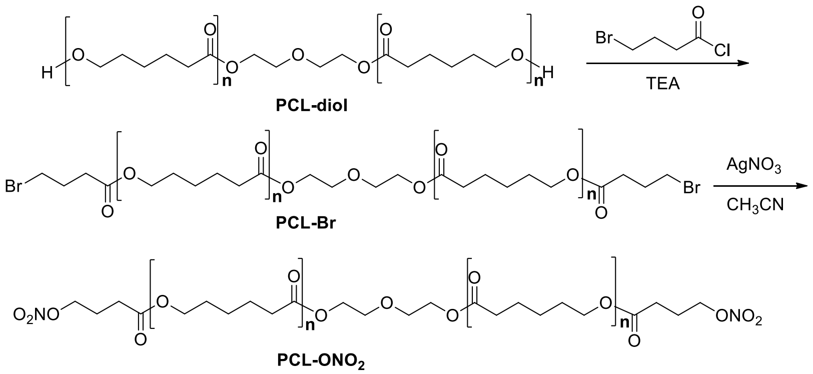

Supplementary Fig. 1. Synthesis of PCL-ONO_2. Synthesis of PCL-ONO_2. To fabricate NO-patch, PCL-ONO_2 was synthesized by acylation of PCL-diol (Mn=2000) with 4-bromobutanoyl chloride, followed by substitution of terminal bromide with $AgNO_3$.

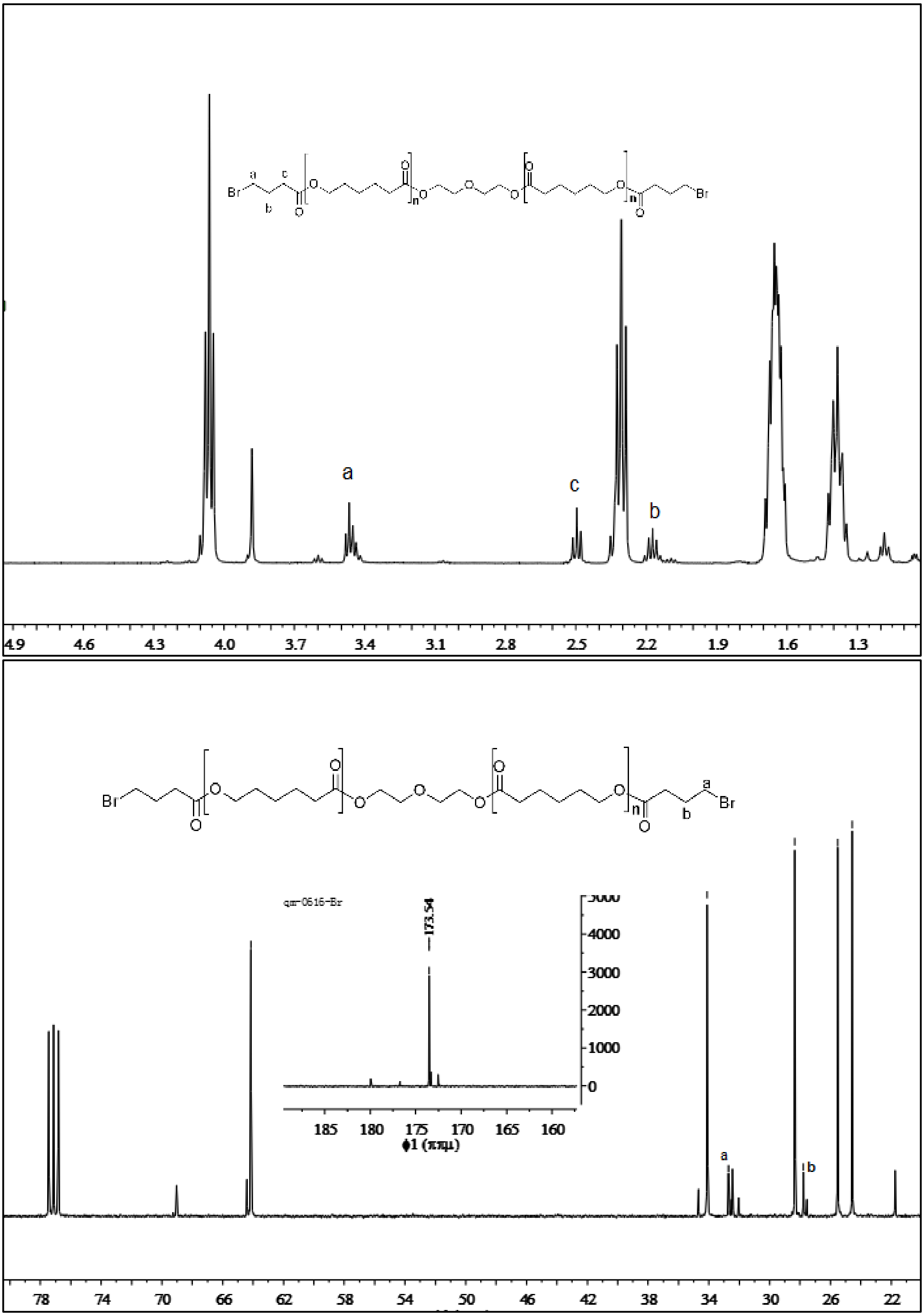

Supplementary Fig. 2. Verification of PCL-Br product. 1H NMR and 13C NMR spectra of PCL-Br was shown.

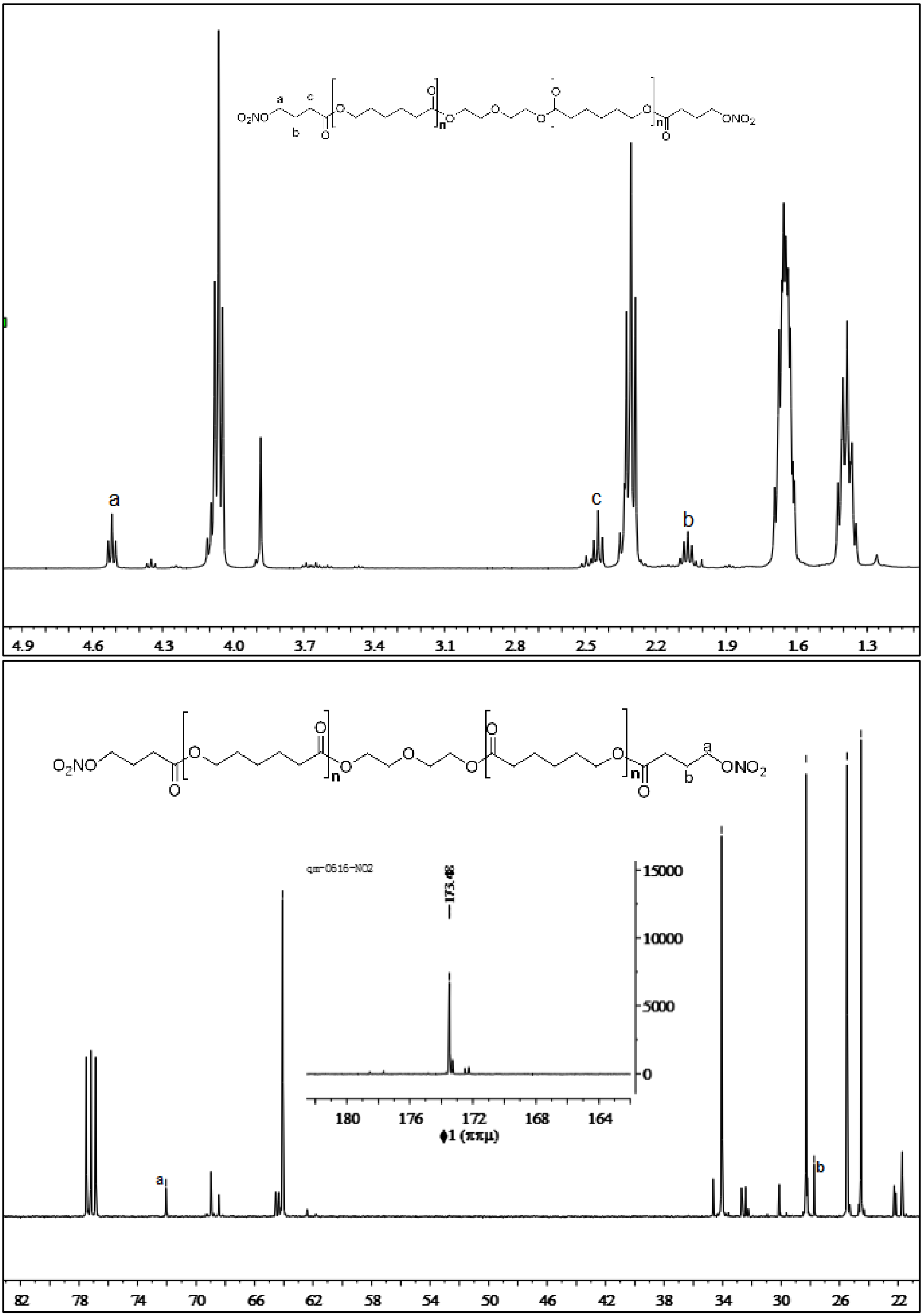

Supplementary Fig. 3. Verification of PCL-ONO2. ^{1}H NMR and ^{13}C NMR spectra of PCL-ONO_2 was shown.

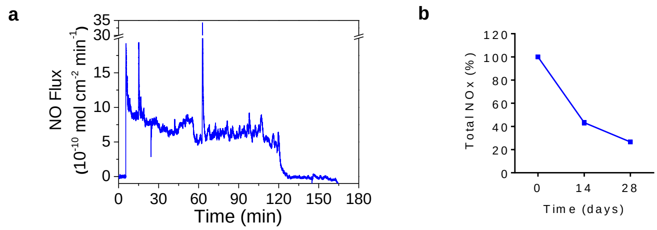

Supplementary Fig. 4. In vivo degradation of NO-patches was evaluated by subcutaneous implantation in rats. Residual NOx in the patch was measured by chemiluminescence. Representative spectrum was shown in (a), and total NOx gradually decreased with implantation time (n=8) (b). NO: nitric oxide.

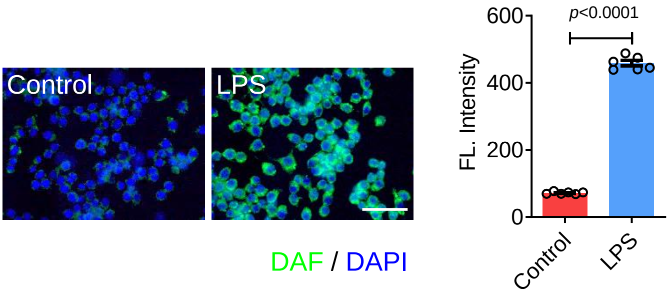

Supplementary Fig. 5. Detection of NO generation by macrophages after LPS stimulation. To detect the generation of NO by macrophages after activation, NO-specific DAF probes was added into the cultures of native macrophages (control) and LPS stimulated macrophages (LPS), and the fluorescence intensity (FL) was measured. Left, representative confocal images showing the generation of NO in activated macrophages, scale bar = 60 μm. Right, the corresponding quantitative results. Data are expressed as mean ± SEM (n=6 independent repeats). Significant difference was detected by unpaired two-sided student t-test. DAF: diaminofluorescein.

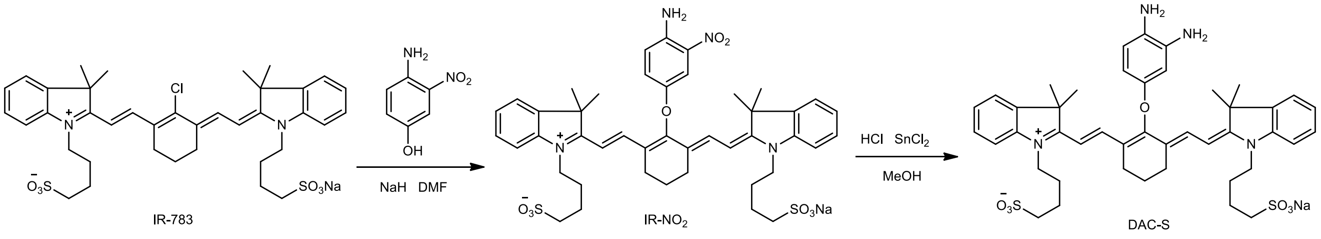

Supplementary Fig. 6. Synthesis of DAC-S. The detailed reaction was described in the Methods of the main text.

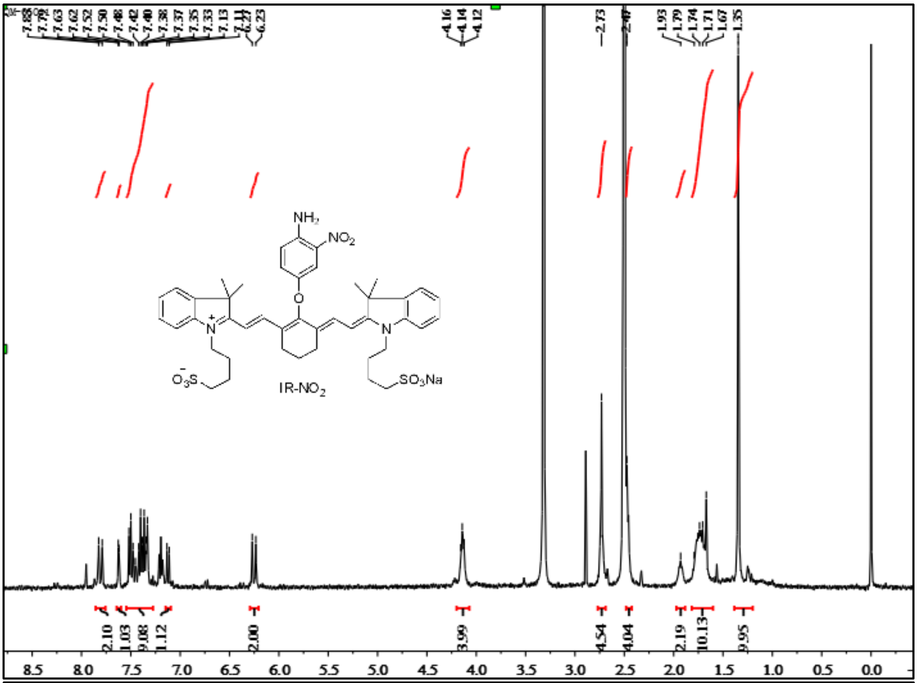

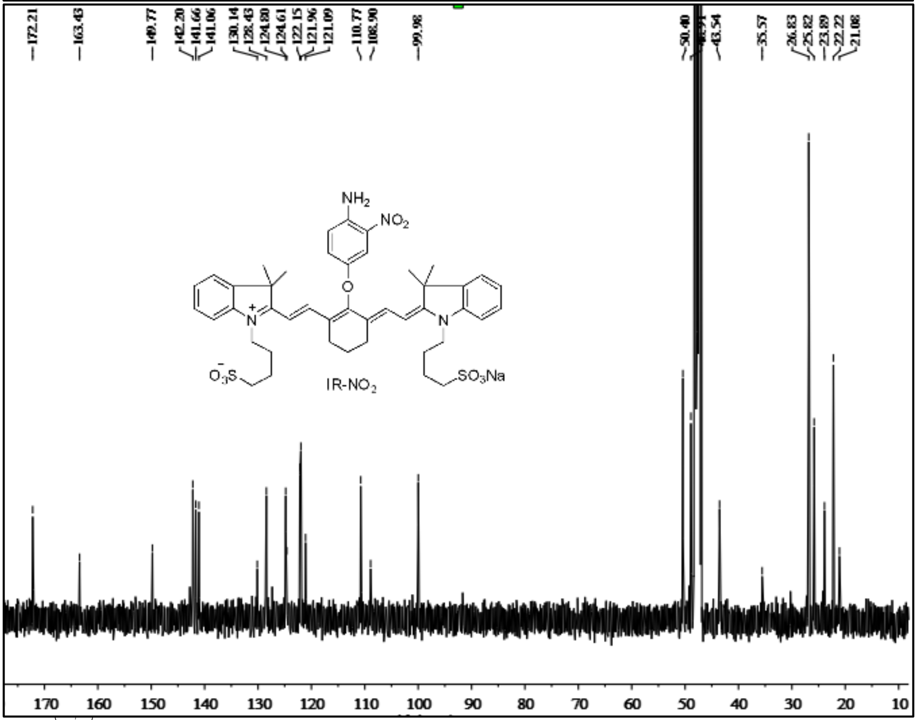

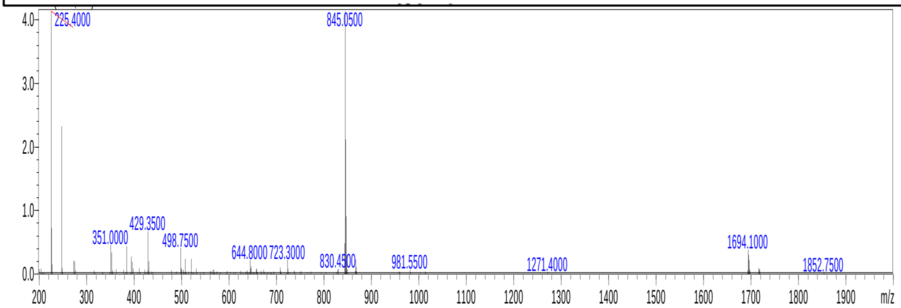

Supplementary Fig. 7. Verification of I-NO$_2$. ^1H NMR, ^{13}C NMR and MS spectra of IR-NO$_2$ was shown.

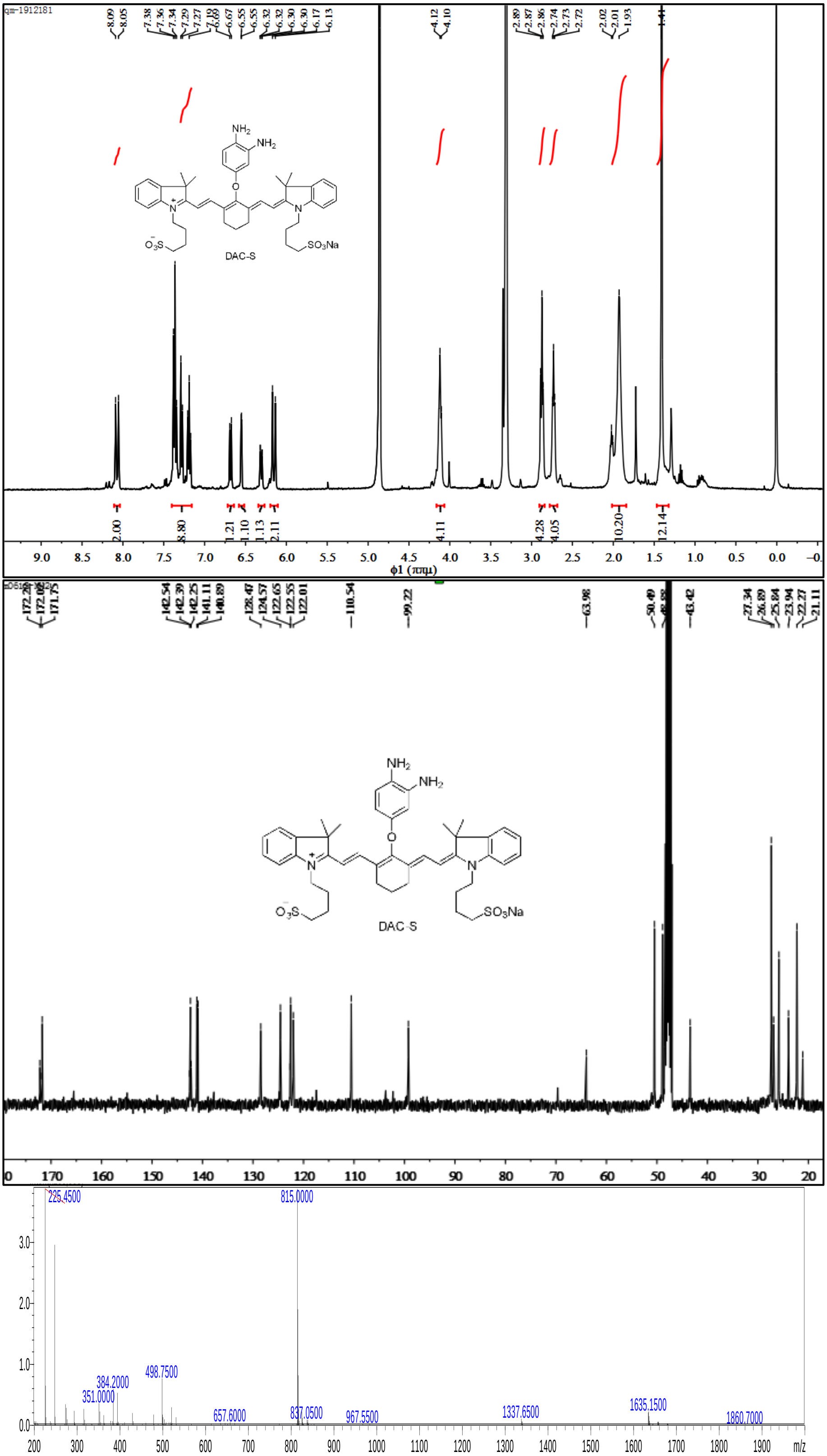

Supplementary Fig. 8. Verification of DAC-S product. ^{1}H NMR, ^{13}C NMR and MS spectra of DAC-S were shown.

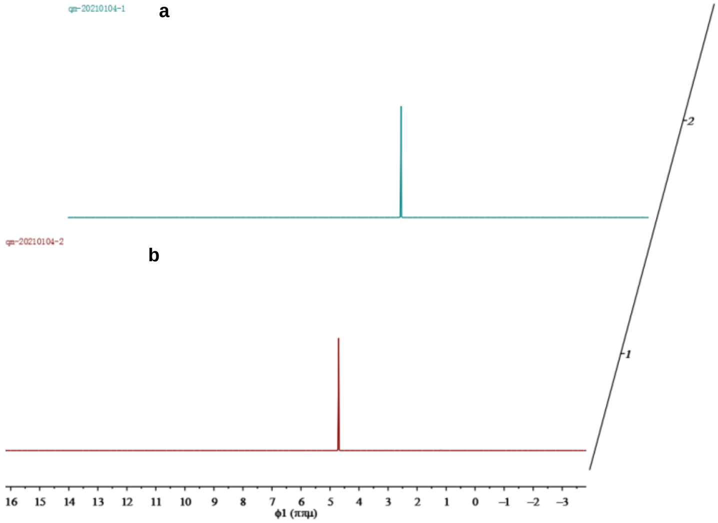

Supplementary Fig. 9. ^{1}H NMR spectra of PCL-ONO_2.^{1}H NMR spectra of PCL-ONO_2 in D_2O before (a) and after degradation in PBS for 3 days (b).

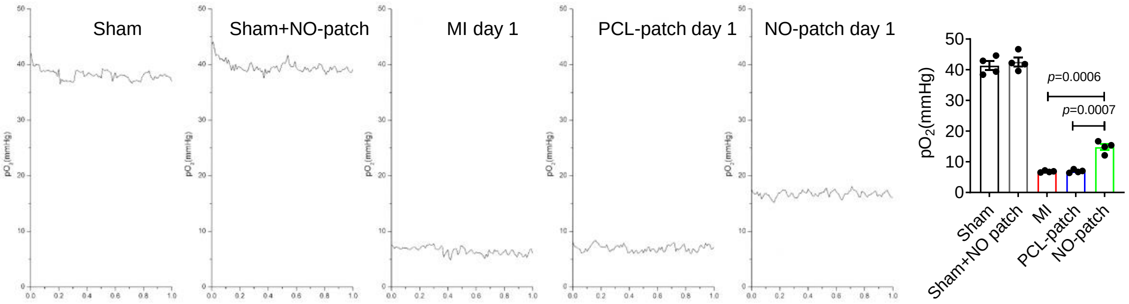

Supplementary Fig. 10. Measurement of cardiac pO2 levels. pO2 levels in sham operated or infarcted myocardium were measured with or without patch treatment. Data are expressed as mean±SEM, n=4 animals for each group. Significant differences were detected by two-tailed one-way ANOVA with Tukey's multiple comparisons test.

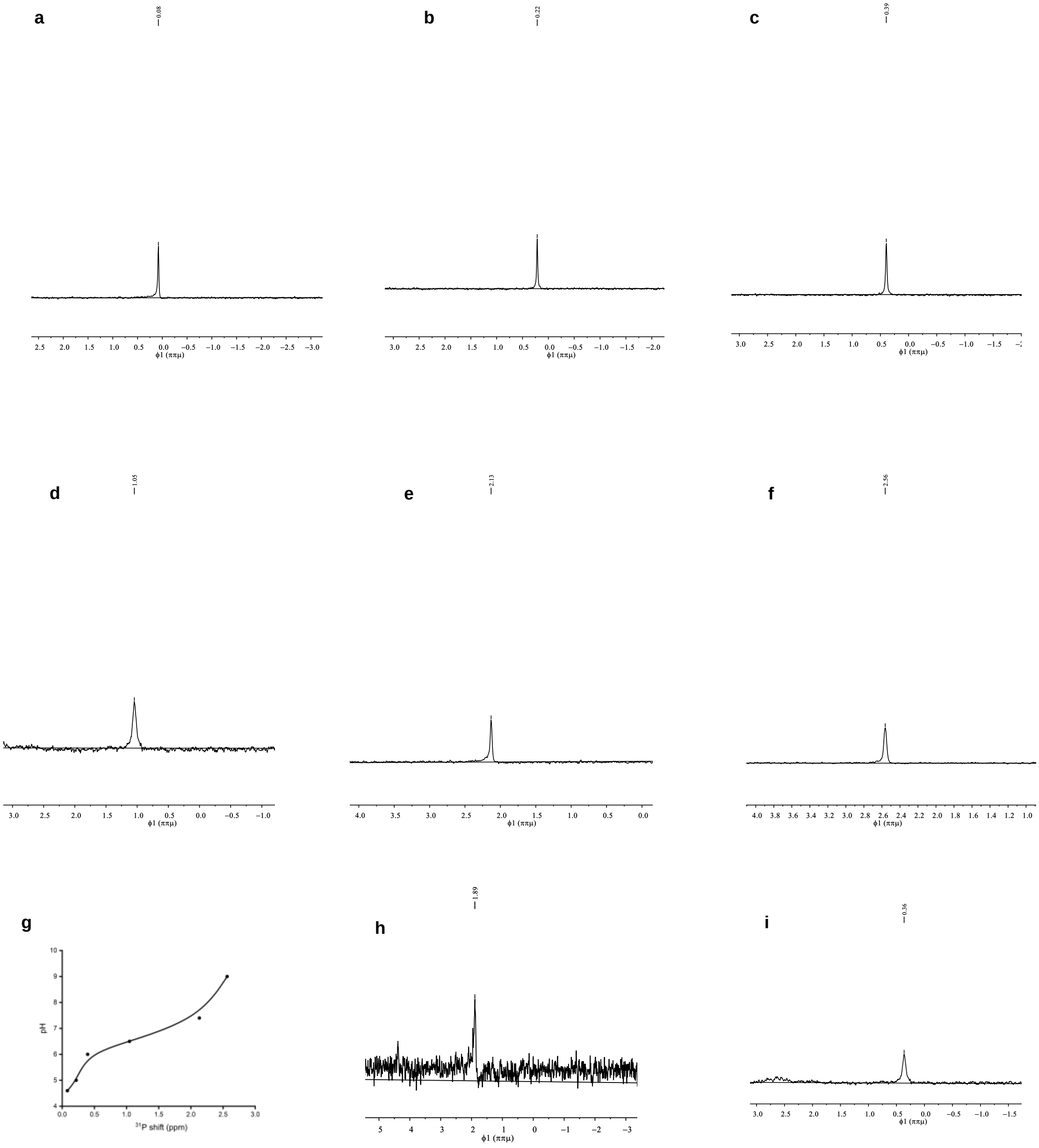

Supplementary Fig. 11. ^{31}P NMR spectra of PBS at different pHs (a-f). (g) Fitted curve between pH with P shift. ^{31}P NMR spectra of heart homogenate from sham (h) and MI groups (i).

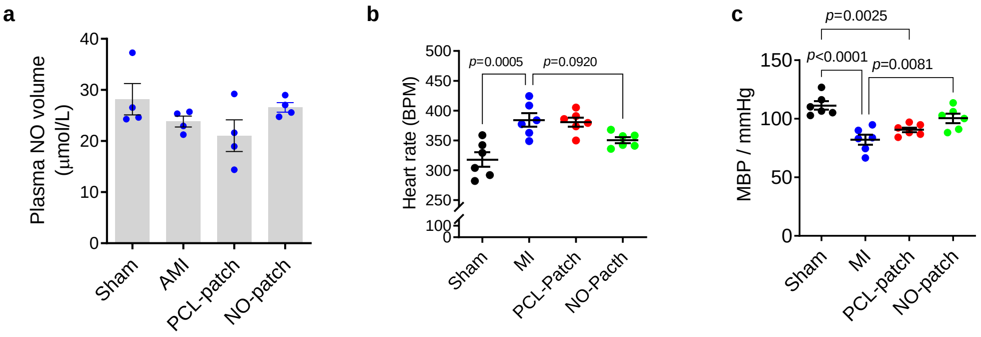

Supplementary Fig. 12. Local NO delivery by NO-patch dose not induce hemodynamic side effects.(a) Plasma NO volume was measured. Data were expressed as mean ± SEM, n=4 animals per group. Heart rate(beat-per-minute, BPM) (b) and mean blood pressure(MBP) (c) were measured in rats. Data were expressed as mean ± SEM, n=6 animals per group. Significant differences were detected by two-tailed one-way ANOVA with Tukey's multiple comparisons test.

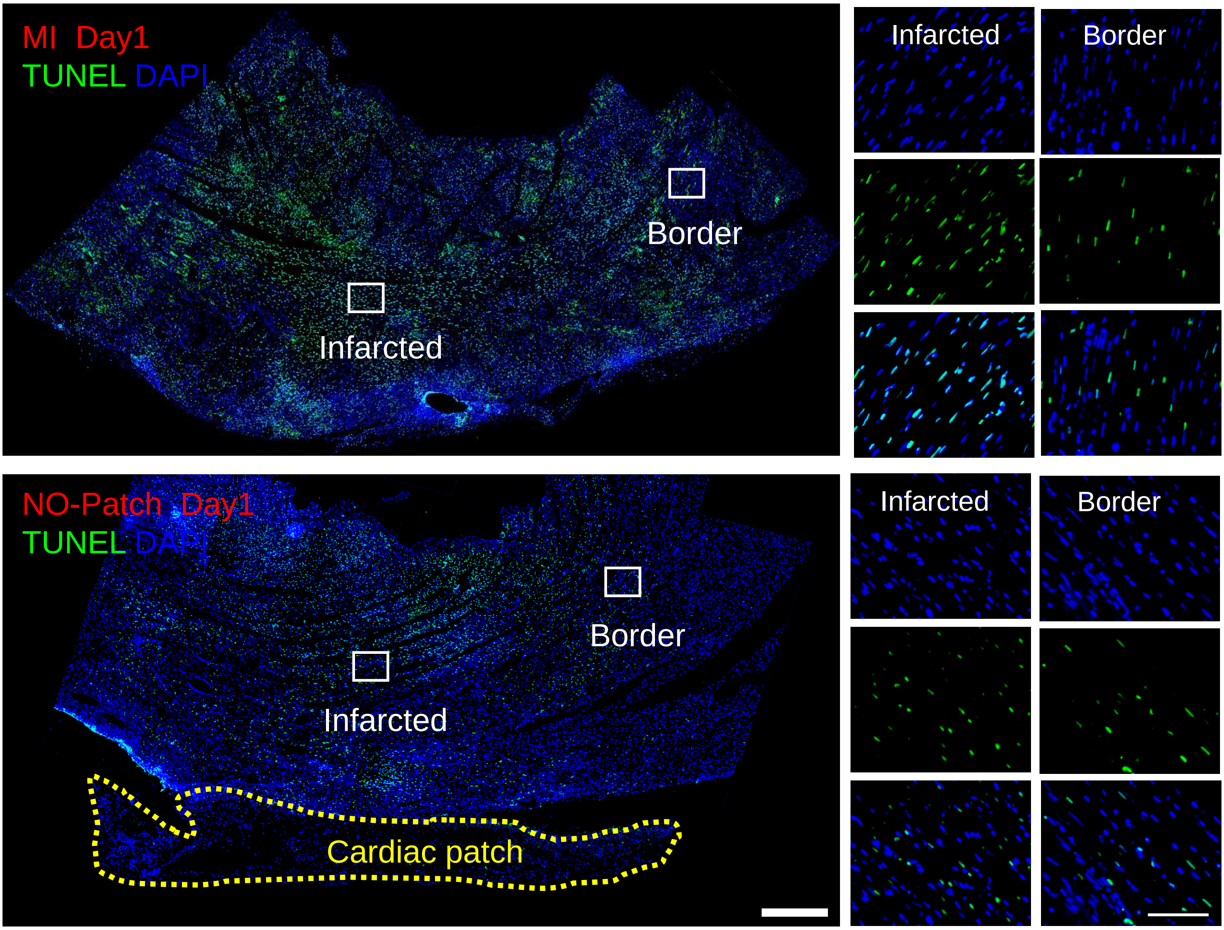

Supplementary Fig. 13. Local NO delivery inhibits cell apoptosis on day 1. Representative images of TUNEL staining show the apoptotic cells in both infarcted and border zones. Scale bar(Left, 1mm; right, 100 μm). This study was repeated in five rats and similar results were acquired.

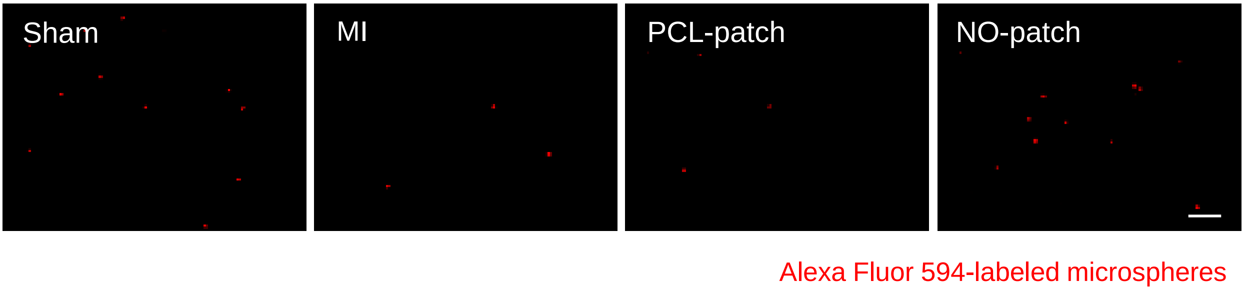

Supplementary Fig. 14. Implantation of NO-patch augmented blood perfusion. Representative images for measurement of myocardium blood flow by detecting the distribution of microspheres in the border zone after intramyocardial perfusion. This study was repeated in three rats and similar results were acquired. Scale bar, 100 μm.

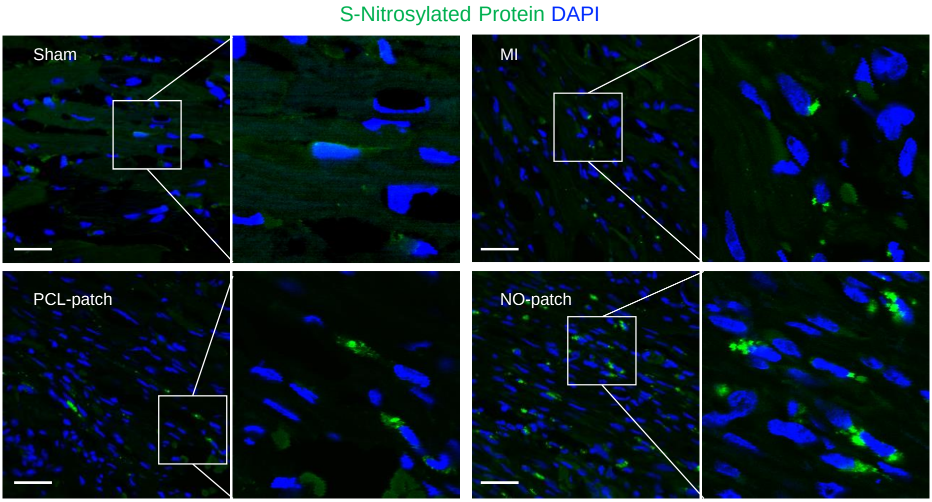

Supplementary Fig. 15. Detection of S-nitrosylated protein in heart tissues one day after patch implantation by using S-Nitrosylated protein detection kit. This study was repeated in four rats and similar results were acquired. Scale bar, 100μm.

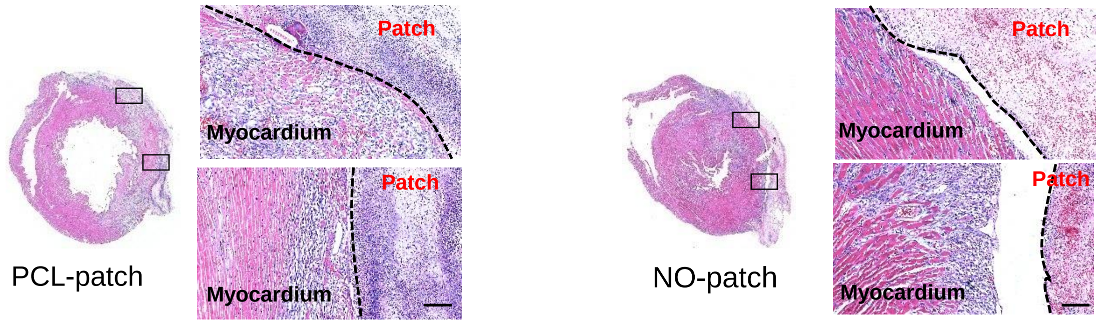

Supplementary Fig. 16. Local NO delivery regulates inflammatory response on day 3. Representative H&E images of the interface between the patch and the myocardium show the infiltration of inflammatory cells. Scale bar, 100 μm. This study was repeated in five rats and similar results were acquired.

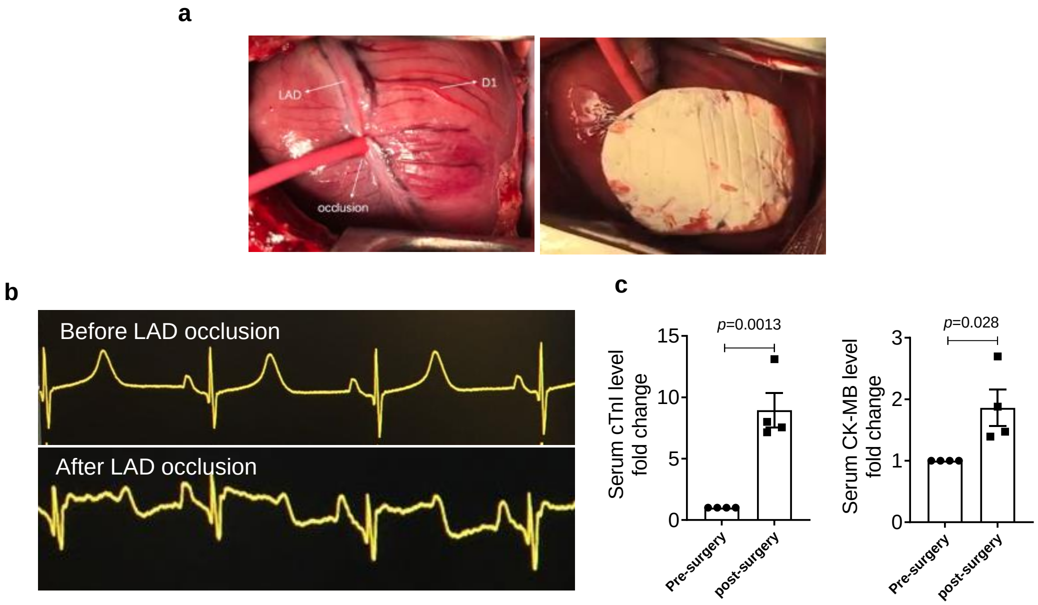

Supplementary Fig. 17. Establishment of pig cardiac I/R injury model. (a) Cardiac patch was implanted to cover infarcted myocardium in a pig model of cardiac ischaemia/reperfusion (I/R). (b) After LAD occlusion, obvious ST-segment elevation was observed in electrocardiograph , confirming the occurrence of MI in pigs. (c) Serum cTnI and CK-MB levels were significantly increased after I/R. Data are expressed as mean $\pm$ SEM, n=4 animals per group. Significant differences were detected by unpaired two-sided student t-test.

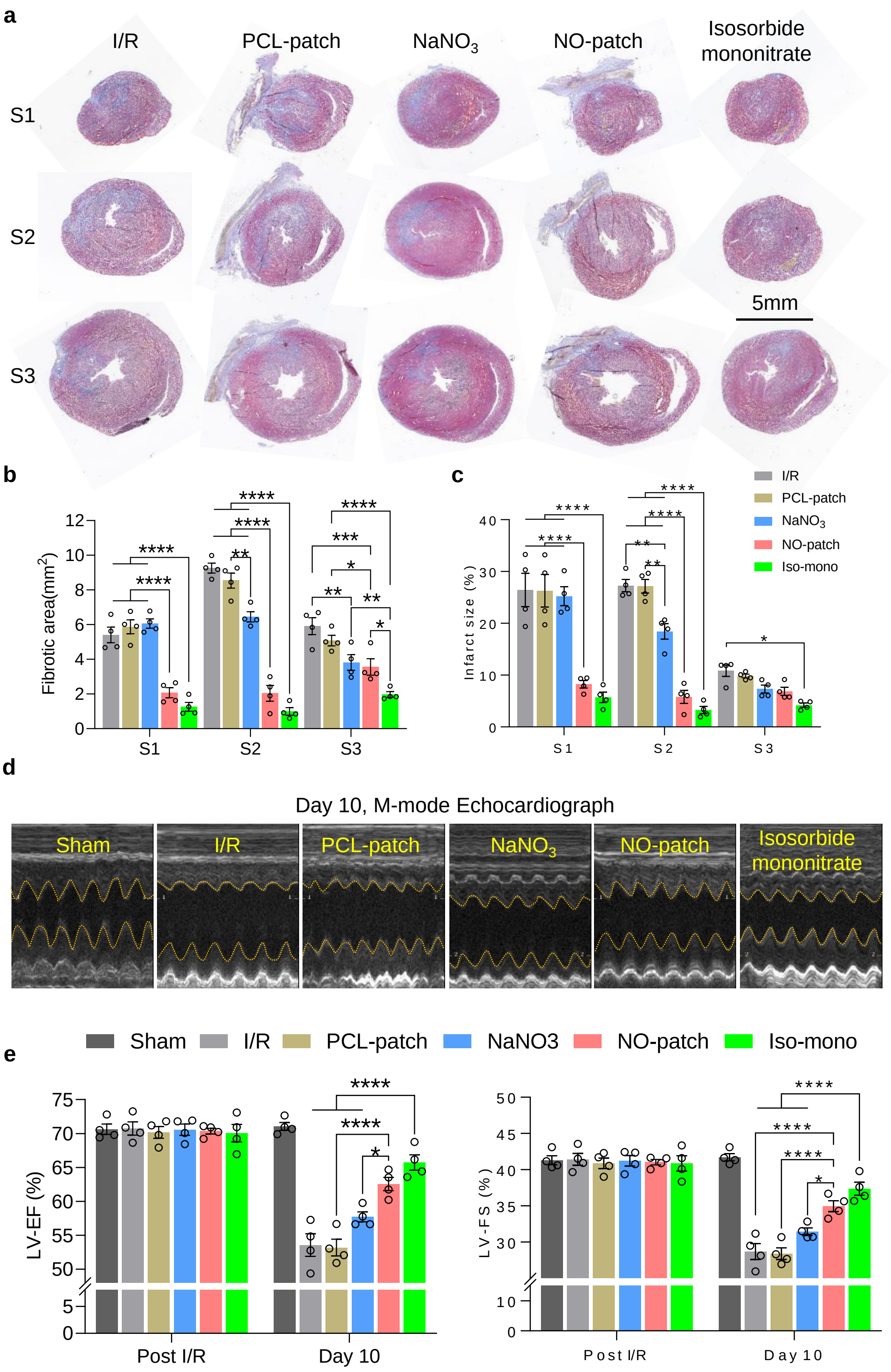

a
I/R
PCL-patch
NaNO3
NO-patch
Isosorbide mononitrate
S1
S2
S3
5mm
b
Fibrotic area(mm2)
0
2
4
6
8
10
12
S1
S2
S3
****
**
*
***
c
I/R
PCL-patch
NaNO3
NO-patch
Iso-mono
Infarct size (%)
0
10
20
30
40
S1
S2
S3
d
Day 10, M-mode Echocardiograph
Sham
I/R
PCL-patch
NaNO3
NO-patch
Isosorbide mononitrate
e
Sham
I/R
PCL-patch
NaNO3
NO-patch
Iso-mono
LV-EF (%)
0
5
50
55
60
65
70
75
Post I/R
Day 10
LV-FS (%)
0
10
30
35
40
45
50
Post I/R
Day 10

Supplementary Fig. 18 Head-to-head comparison of the therapeutic efficacy after treatment by NO-patch implantation, oral administration of isosorbide mononitrate or sodium nitrate in a rat model of cardiac ischemia/reperfusion heart injury at 10 days. (a) Masson staining shows the infarct, and accordingly, the infarct area (b) and the ratio of infarct area to the whole left ventricular area(infarct size, c) were measured. Scale bar, 5mm. Data were expressed as mean ± SEM, n=4 animals per group. (b) p=0.0017(S2, PCL-patch vs. $NaNO_3$), p=0.0003 (S3, I/R vs. NO-patch), p=0.0017 (S3, I/R vs. $NaNO_3$), p=0.0367 (S3, PCL-patch vs. NO-patch), p=0.0268 (S3, NO-patch vs. Iso-mono), p=0.0069 (S3, Iso-mono vs. $NaNO_3$), ****p<0.0001. (c) p=0.0016 (S2, I/R vs. $NaNO_3$), p=0.0018 (S2, PCL-patch vs. $NaNO_3$), p=0.0287 (S3, I/R vs. Iso-mono). Significant differences were detected by two-way ANOVA with Tukey's multiple comparisons test. (d) Representative M-mode echocardiograph images. (e) Cardiac ejection function was measured immediately after reperfusion (Post I/R) and 10 days after treatment; the left ventricular ejection fraction(LV-EF) and left ventricular fraction shortening(LV-FS) were calculated. Data were expressed as mean ± SEM, n=4 animals per group. Significant differences were detected by two-way ANOVA with Šídák's multiple comparisons test. *p<0.05, **p<0.01, ***p<0.001, ****p<0.0001, p=0.0467 (LV-EF, day 10, NO-patch vs. $NaNO_3$), p=0.0383 (LV-FS, day 10, NO-patch vs. $NaNO_3$).
