# Supplementary material for: Nitrate-functionalized patch confers cardioprotection and improves heart repair after myocardial infarction via local nitric oxide delivery
Source: Nat Commun. 2021 Jul 23;12:4501. doi: 10.1038/s41467-021-24804-3 (PMC8302626; doi:10.1038/s41467-021-24804-3)
Supplement: Supplementary file 3 — Reporting Summary [file 41467_2021_24804_MOESM3_ESM.pdf]

nature research

Corresponding author(s): Qiang Zhao

Last updated by author(s): Jun 6, 2021

Reporting Summary

Nature Research wishes to improve the reproducibility of the work that we publish. This form provides structure for consistency and transparency in reporting. For further information on Nature Research policies, see our Editorial Policies and the Editorial Policy Checklist.

Statistics

For all statistical analyses, confirm that the following items are present in the figure legend, table legend, main text, or Methods section.

n/a	Confirmed	
☐	☒	The exact sample size (*n*) for each experimental group/condition, given as a discrete number and unit of measurement
☐	☒	A statement on whether measurements were taken from distinct samples or whether the same sample was measured repeatedly
☐	☒	The statistical test(s) used AND whether they are one- or two-sided<br>*Only common tests should be described solely by name; describe more complex techniques in the Methods section.*
☒	☐	A description of all covariates tested
☐	☒	A description of any assumptions or corrections, such as tests of normality and adjustment for multiple comparisons
☐	☒	A full description of the statistical parameters including central tendency (e.g. means) or other basic estimates (e.g. regression coefficient) AND variation (e.g. standard deviation) or associated estimates of uncertainty (e.g. confidence intervals)
☐	☒	For null hypothesis testing, the test statistic (e.g. *F*, *t*, *r*) with confidence intervals, effect sizes, degrees of freedom and *P* value noted<br>*Give P values as exact values whenever suitable.*
☒	☐	For Bayesian analysis, information on the choice of priors and Markov chain Monte Carlo settings
☒	☐	For hierarchical and complex designs, identification of the appropriate level for tests and full reporting of outcomes
☒	☐	Estimates of effect sizes (e.g. Cohen's *d*, Pearson's *r*), indicating how they were calculated

Our web collection on statistics for biologists contains articles on many of the points above.

Software and code

Policy information about availability of computer code

Data collection	Image J version 1.8.0, Zeiss Axio Imager M2 Advanced Microscope Platform, CRI Maestro noninvasive fluorescence imaging system, Circle CVI42 Calgary software, OxyLite monitoring System, Excel 2016(Microsoft), Vevo 2100 Imaging System(FujiFilm VisualSonics), Image Pro Plus 6.0.
Data analysis	Cardiac functional measurements were acquired with Vevo 2000 to generate statistical data; Histological staining images were acquired with Zeiss Axio Imager or FV-31s-SW software, and the quantitative data were analyzed by Image J or Image Pro Plus to generate statistical data. , All statistical datasets were analyzed using Prism 7 (GraphPad) software

For manuscripts utilizing custom algorithms or software that are central to the research but not yet described in published literature, software must be made available to editors and reviewers. We strongly encourage code deposition in a community repository (e.g. GitHub). See the Nature Research guidelines for submitting code & software for further information.

Data

Policy information about availability of data

All manuscripts must include a data availability statement. This statement should provide the following information, where applicable:

- Accession codes, unique identifiers, or web links for publicly available datasets
- A list of figures that have associated raw data
- A description of any restrictions on data availability

The source data underlying Figs. 1c-d, 2c-d, 3b-f, 4a-d, 5a-d, 6a, 6c-g, 7c-e, 8b-d, 9b-c, 9e-g , Supplementary Table 1, and Supplementary Figs. 4, 5, 10, 12, 17 and 18 are provided as a Source Data file, and the datasets that support the findings of this study are available from the corresponding author upon reasonable request.

Field-specific reporting

Please select the one below that is the best fit for your research. If you are not sure, read the appropriate sections before making your selection.

- [x] Life sciences
- [ ] Behavioural & social sciences
- [ ] Ecological, evolutionary & environmental sciences

For a reference copy of the document with all sections, see nature.com/documents/nr-reporting-summary-flat.pdf

Life sciences study design

All studies must disclose on these points even when the disclosure is negative.

Sample size	No statistical methods were used to predetermine the sample size. For all these experiments, at least 3 independent repeats/animals were deployed in each group, which allowed sufficient statistics to perform unpaired student t-test or ANOVA analysis, and gave p values to indicate the significance. To proceed with statistical analysis, more than three animals or repeats were performed for each test.
Data exclusions	No data was excluded.
Replication	All experiments were repeated from at least three independent tests, and all attempts at replication were successful.
Randomization	All samples was randomly assigned, and analyzed together in each experiment.
Blinding	Data acquisition and analysis were performed by investigators who are blinded to the groups.

Reporting for specific materials, systems and methods

We require information from authors about some types of materials, experimental systems and methods used in many studies. Here, indicate whether each material, system or method listed is relevant to your study. If you are not sure if a list item applies to your research, read the appropriate section before selecting a response.

Materials & experimental systems

n/a	Involved in the study	
[ ]	[x]	Antibodies
[ ]	[x]	Eukaryotic cell lines
[x]	[ ]	Palaeontology and archaeology
[ ]	[x]	Animals and other organisms
[x]	[ ]	Human research participants
[x]	[ ]	Clinical data
[x]	[ ]	Dual use research of concern

Methods

n/a	Involved in the study	
[x]	[ ]	ChIP-seq
[x]	[ ]	Flow cytometry
[x]	[ ]	MRI-based neuroimaging

Antibodies

Antibodies used	Anti-Sarcomeric Alpha Actinin(a-SA) antibody [EA-53] (Abcam, ab9465, 1:100), CD31 (Abcam, ab28364, 1:100), Anti-α smooth muscle actin(a-SMA) Antibody(monoclonal,1A4) (Boster, BM0002, 1:500), Anti-CD68 antibody [C68/684] (Abcam, ab201340, 1:100), Anti-CD86 antibody [BU63] (Abcam, ab234000, 1:100), CD206 (Abcam, ab64693, 1:100), Recombinant Anti-Ki67 antibody [SP6] (Abcam, ab16667, 1:200), Alexa Fluor 594-conjugated goat anti-mouse IgG (Abcam, ab150116, 1:200), and Alexa Fluor 488-conjugated goat anti-rabbit IgG (Abcam, ab150077, 1:200)
Validation	All antibodies were purchased after referring to the reactivity and species on manufacturer's website. By referring to their reactivity to mouse, rat, as well as application in immunofluoresence staining,these antibodies were ordered. All the antibodies used in this study work excellently.<br>Anti-Sarcomeric Alpha Actinin antibody [EA-53] (ab9465):https://www.abcam.com/sarcomeric-alpha-actinin-antibody-ea-53-ab9465.html<br>Anti-CD31 antibody (ab28364): https://www.abcam.com/cd31-antibody-ab28364.html<br>Anti-α smooth muscle actin(a-SMA) Antibody(monoclonal,1A4): http://www.boster.com.cn/product/anti-smooth-muscle-actin-a-sma-antibody-monoclonal-1a4_bm0002.html<br>Anti-CD68 antibody [C68/684] (ab201340): https://www.abcam.com/cd68-antibody-c68684-ab201340.html<br>Anti-CD86 antibody [BU63] (ab234000): https://www.abcam.com/cd86-antibody-bu63-ab234000.html<br>Anti-Mannose Receptor(CD206) antibody (ab64693): https://www.abcam.com/mannose-receptor-antibody-ab64693.html<br>Recombinant Anti-Ki67 antibody [SP6] (ab16667): https://www.abcam.com/ki67-antibody-sp6-ab16667.html<br>Goat Anti-Mouse IgG H&L (Alexa Fluor® 594) (ab150116): https://www.abcam.com/goat-mouse-igg-hl-alexa-fluor-594-ab150116.html<br>Goat Anti-Rabbit IgG H&L (Alexa Fluor® 488) (ab150077): https://www.abcam.com/goat-rabbit-igg-hl-alexa-fluor-488-ab150077.html

Eukaryotic cell lines

Policy information about cell lines

Cell line source(s)	Macrophage (Raw 264.7) were purchased from the American Type Culture Collection (ATCC, USA).
Authentication	Macrophage was tested by flowcytometry for expression of H-2d, but was negative for surface immunoglobulin (sIg), Ia (Ia) and Thy-1.2 (Thy-1.2).
Mycoplasma contamination	Mycoplasma testing (PCR method) is routinely performed in the lab, and all cell lines tested negative for mycoplasma contamination.
Commonly misidentified lines (See ICLAC register)	No misidentified cell lines were used in the study.

Animals and other organisms

Policy information about studies involving animals; ARRIVE guidelines recommended for reporting animal research

Laboratory animals	Sprague-Dawley (SD) rats (8-weeks old, male) and Bama mini-pigs (4 weeks old, male) with mean weight of 12 kg were used in study. The details can be found in the Methods section.
Wild animals	No wild animal was used in this study.
Field-collected samples	This study did not involve field-collected samples.
Ethics oversight	All procedures for animal studies were approved by the Committee of Animal Use and Care of Nankai University and the Ethics Committee of Shanghai Jiaotong University Animal Department.

Note that full information on the approval of the study protocol must also be provided in the manuscript.
